# Letter to the editor

**DOI:** 10.1186/s40662-021-00270-2

**Published:** 2021-12-10

**Authors:** Miguel Faria Ribeiro

**Affiliations:** Center of Physics, University of Minho, Braga, Portugal

## Dear Editor,

Recently, Alió and co-works published a study [1] comparing the clinical optical image quality of patients following implantation with different intraocular lenses (IOLs), based on the analysis of the wavefront measured with a pyramidal wavefront sensor-based aberrometer. A set of nine groups of patients was evaluated, including patients implanted with two diffractive trifocals.

**Fig. 1:**
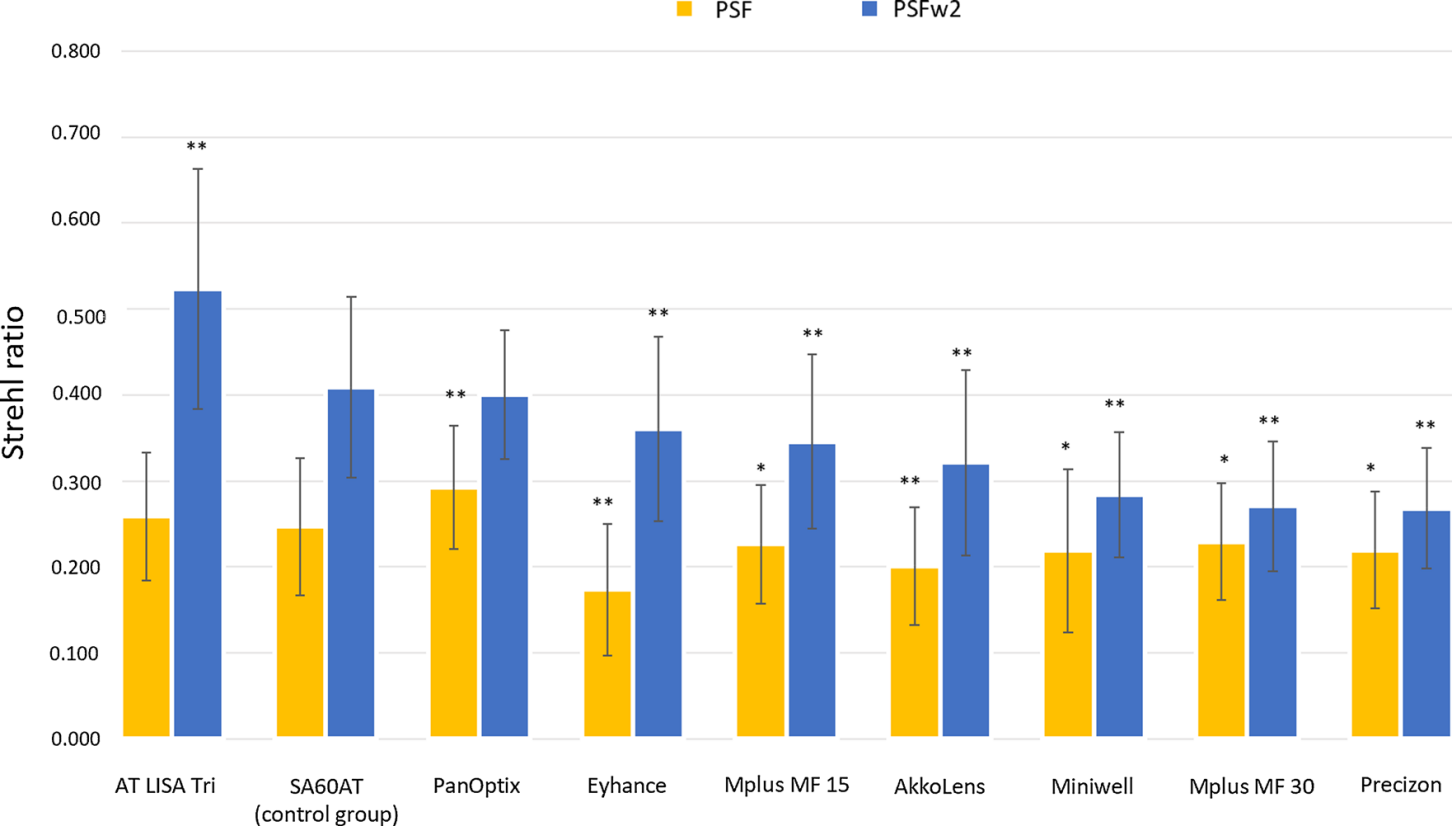
Depicted from Alió et al. [1]. PSF Strehl ratio with and without low-order aberration for each group, obtained with a pyramidal wavefront sensor-based aberrometer, and level of significance compared to the monofocal spherical control group. *P < 0.05*,* **P < 0.001

Surprisingly, accordingly to the results reported by the authors, the group implanted with the trifocal diffractive AT Lisa stands out in terms of distance image quality calculated from the captured wavefront, with a monochromatic Strehl ratio above other groups implanted with monofocal designs. Another example of these intriguing result is the similarity in Strehl ratio between the spherical monofocal control group and the group implanted with the PanOptix trifocal. Considering that any residual refraction was accounted in the analysis and that other contributions apart from the IOL design such as patient high-order aberrations, lens decentration or tilt were equally distributed between groups, the results presented in Fig. 1 are likely caused by an erroneous interpretation of the wavefront data.

The authors claim that due to its high resolution (41 µm) the pyramidal wavefront sensor is capable of overcoming the limitations imposed by Hartmann-Shackman sensor-based aberrometers, where for lenslet locations away from the centre of the IOL multiple diffractive zones can cross through the aperture of the same lenslet producing multiple spot patterns [2–4]. Although it could be possible that the finer sampling of the pyramidal wavefront sensor might allow it to discriminate small adjacent parts of the wavefront with abrupt changes in slope, the authors do not seem to consider the wavelength dependency of these diffractive elements [2–4]. Clinical based aberrometers normally rely on infrared radiation to reduce patient discomfort and minimize pupil constriction. Although several papers that deal with this problem are cited by Alió et al., this limitation does not seem to be taken in to consideration leading to the erroneous interpretations plotted in Fig. 1.

**Fig. 2:**
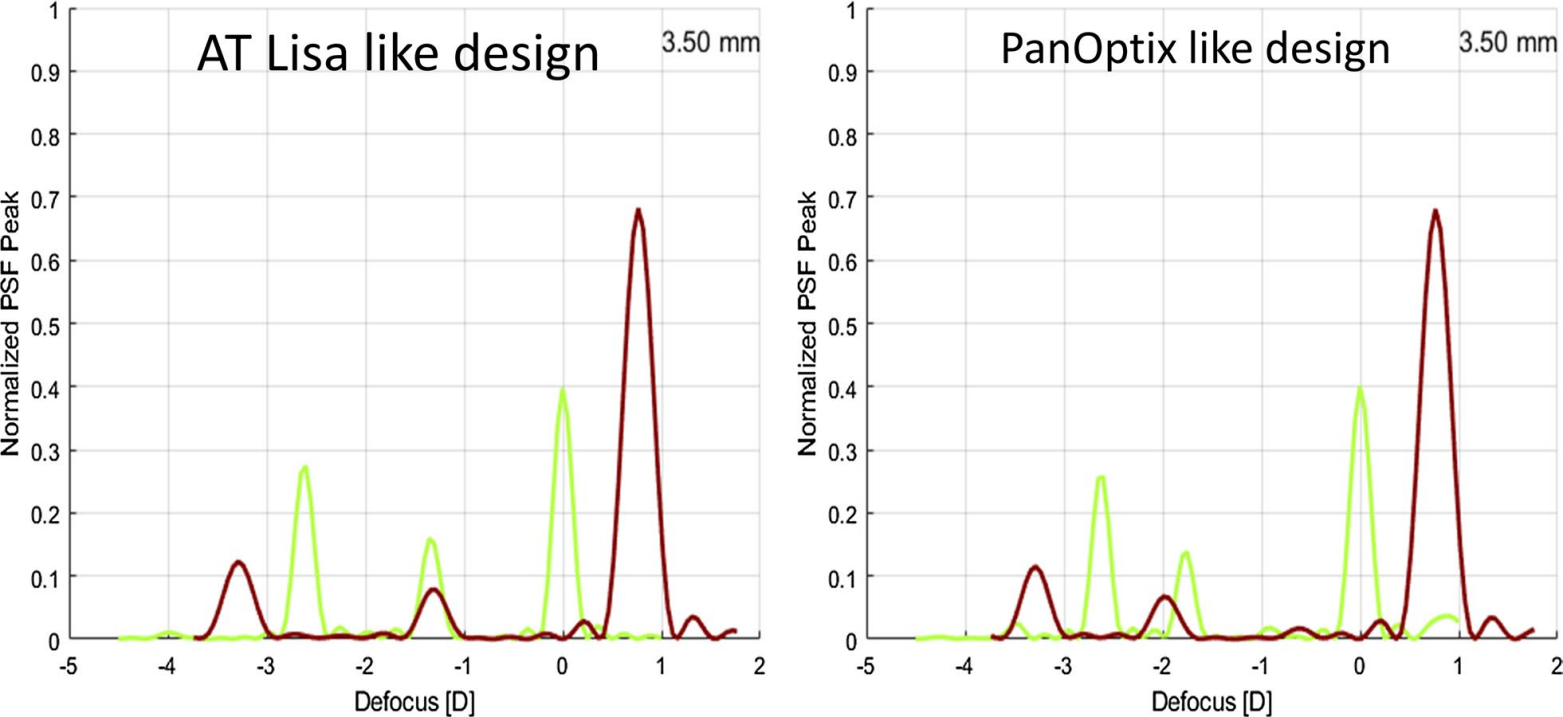
Normalized light distribution calculated for 550 nm (green line) and 850 nm (red line), for two diffractive designs similar to the AT Lisa (left) and to the PanOptix (right) IOLs. Dispersion was modelled using Cornu’s hyperbolic formula, with coefficients fitted for a pseudo phakic eye [LCA = 1.667–591/(wavelength − 195.9)], were LCA stands for longitudinal chromatic aberration, in dioptres, and wavelength is expressed in meters

The infrared radiation used by pyramidal wavefont sensor (850 nm) introduces two major important modifications to the diffractive wavefronts captured in the instruments CCD. First, the diffractive effective addition power will be higher for the longer wavelength since diffractive power is linearly dependent on wavelength. Second, since the step heights of the diffractive profile are normally optimized for 550 nm, light distribution will be unbalanced for infrared radiation with most of the constructive interference occurring at the distance focus [2–5]. These two effects are illustrated in Fig. 2.

The correction applied due to the longitudinal chromatic aberration of the eye will only correct the best focus shift seen at 850 nm (Fig. 2) and not for the redistribution of light [5]. Thus, even if the sampling of the pyramidal wavefront sensor is high enough to capture the diffractive wavefront with enough detail, PSFs and MTFs based on these measurements will therefore be overly optimistic for distance vision [2–4] and not representative of the real visual function of the patients implanted with these IOLs.

## Data Availability

Not applicable.
